# Proximal iteratively reweighted algorithm for low-rank matrix recovery

**DOI:** 10.1186/s13660-017-1602-x

**Published:** 2018-01-08

**Authors:** Chao-Qun Ma, Yi-Shuai Ren

**Affiliations:** Business School, Hunan University, Changsha, 410082 China

**Keywords:** compressed sensing, matrix rank minimization, reweighted nuclear norm minimization, Schatten-*p* quasi-norm minimization

## Abstract

This paper proposes a proximal iteratively reweighted algorithm to recover a low-rank matrix based on the weighted fixed point method. The weighted singular value thresholding problem gains a closed form solution because of the special properties of nonconvex surrogate functions. Besides, this study also has shown that the proximal iteratively reweighted algorithm lessens the objective function value monotonically, and any limit point is a stationary point theoretically.

## Introduction

The low-rank matrix recovery problem has been a research hotpot recently [1, 2], and it has a range of applications in many fields such as signal or image processing [3, 4], subspace segmentation [5], collaborative filtering [6], and system identification [7]. Matrix rank minimization under affine equality constraints is generally formulated as follows:

1.1 $$ \min_{X} \operatorname{rank} ( X ) \quad\text{s.t.}\quad \mathrm{A} ( X ) = b, $$

where the linear map $\mathrm{A}:R^{m \times n} \to R^{P}$ and the vector *b* are known.

Unfortunately, solving the above rank minimization problem (1.1) directly is an NP-hard problem [8], thus this problem is computationally infeasible. Therefore, the convex relations of these methods have been proposed and studied in the literature. For example, Recht *et al*. [8] proposed a nuclear norm minimization method for the matrix reconstruction. The tightest convex relaxation of problem (1.1) is the following nuclear norm minimization problem:

1.2 $$ \min_{X} \Vert X \Vert _{\bigstar} \quad\text{s.t.} \quad\mathrm{A} ( X ) = b, $$

where $\Vert X \Vert _{\bigstar} = \sum_{i = 1}^{r} \sigma_{i} ( X )$ is the sum of all the singular values of $X \in R^{m \times n}$ with $\operatorname{rank} ( X ) = r$ (without loss of generality, $n \le m$). It has been shown that problem (1.2) shares common solutions with problem (1.1) under some sufficient conditions (see, *e.g.*, [8, 9]).

However, the exact recovery of the low-rank matrix requires more measurements via nuclear norm minimization. Recently, some experimental observations and theoretical guarantees have shown the superiority of $\ell_{p}$ quasi-norm minimization to $\ell_{1}$ minimization in compressive sampling [10]. Therefore, the $\ell_{p}$ quasi-norm minimization [11–13] was introduced instead of the nuclear norm minimization in order to give a better approximation to the original problem (1.1). Therefore, the $\ell_{p}$ quasi-norm minimization can be formulated as

1.3 $$ \min_{X} \Vert X \Vert _{p}^{p}\quad \text{s.t.}\quad \mathrm{A} ( X ) = b, $$

where $\Vert X \Vert _{p} = ( \sum_{i = 1}^{r} \sigma_{i}^{p} ( X ) )^{1/p}$ for some $p \in ( 0,1 )$.

However, in practice, the observed data in the low-rank matrix recovery problem may be contaminated with noise, namely $b = \mathrm{A}X + e$, where *e* contains measurement errors dominated by certain normal distribution. In order to recover the low-rank matrix robustly, problem (1.3) can be modified to

1.4 $$ \min_{X} \Vert X \Vert _{p}^{p} \quad\text{s.t.}\quad \bigl\Vert \mathrm{A} ( X ) - b \bigr\Vert _{2} \le \varepsilon, $$

where $\Vert \cdot \Vert _{2}$ is the $\ell_{2}$ norm of vector and $\varepsilon\ge \Vert e \Vert _{2}$ is some constant.

Under some conditions, problems (1.3) and (1.4) can be rewritten as the following unconstrained model:

1.5 $$ \min_{X} \tau \Vert X \Vert _{p}^{p} + \frac{1}{2} \bigl\Vert \mathrm{A} ( X ) - b \bigr\Vert _{2}^{2}, $$

where $\tau> 0$ is a given parameter. Since the above problem (1.5) is nonconvex and NP-hard, thus the researchers throughout the world proposed and analyzed some iterative reweighted algorithms [13–15]. The key idea of the iterative reweighted technique is to solve a convex problem with a given weight at each iteration and update the weight at every turn.

Different from previous studies, based on the weighted fixed point method, this paper puts forward a proximal iteratively reweighted algorithm to recover a low-rank matrix. Due to the special properties of nonconvex surrogate functions, the algorithm iteratively has a closed form solution to solve a weighted singular value thresholding problem. Also, in theory, this study has proved that the proximal iteratively reweighted algorithm decreases the objective function value monotonically, and any limit point is a stationary point.

The remainder of this paper is organized as follows. Section 2 introduces some notations and preliminary lemmas, and Section 3 describes the main results. The conclusion is followed in Section 4.

## Preliminaries

Recently, Lai *et al*. [13] considered the following unconstrained problem:

2.1 $$ \min_{X} F ( X ) = \tau \operatorname{tr} \bigl( \bigl( X^{T}X + \varepsilon I \bigr)^{p/2} \bigr) + \frac{1}{2} \bigl\Vert \mathrm{A} ( X ) - b \bigr\Vert _{2}^{2}, $$

where *I* is the $n \times n$ identity matrix and $\varepsilon> 0$ is a smoothing parameter. By the definition in [13], we have

2.2 $$ \operatorname{tr} \bigl( \bigl( X^{T}X + \varepsilon I \bigr)^{p/2} \bigr) = \sum_{i = 1}^{n} \bigl( \sigma_{i} ( X )^{2} + \varepsilon \bigr)^{p/2}. $$

### Lemma 2.1

([16])

*Let* $\varphi ( X ) = \psi \circ\sigma ( X ) = \sum_{i = 1}^{n} ( \vert \sigma_{i} ( X ) \vert + \varepsilon )^{p}$, *where the function* $\varphi: \mathbb{R}^{m \times n} \to [ - \infty, + \infty ]$ *with* $n \le m$ *is orthogonally invariant*; $\psi: \mathbb{R}^{m \times n} \to [ - \infty, + \infty ]$ *is an absolutely symmetric function and* $p \in ( 0,1 )$, *then* $\varphi= \psi\circ\sigma$ *is subdifferentiable at matrix* $X \in\mathbb{R}^{m \times n}$ *and*

$$\partial\varphi ( X ) = pU\operatorname{Diag} \biggl\{ \frac{c_{i}}{ ( \sigma_{i} ( X ) + \varepsilon )^{1 - p}}:i \in\Omega \biggr\} V^{T} $$

*with* $X = U\Sigma V^{T}$ *being the* *SVD* *of* *X*, *and*

$$c_{i} = \left \{ \textstyle\begin{array}{l@{\quad}l} 1, &\sigma_{i} ( X ) > 0, \\ {[ - 1,1 ]}, &\sigma_{i} ( X ) = 0, \end{array}\displaystyle \right . $$

*is a constant depending only on the value of* $\sigma_{i} ( X )$ *for each* $i \in\Omega$.

From Lemma 2.1, let $m = n$ and the matrix *Y* be a semidefinite matrix, then $Y = Y^{T}$ and the subdifferentiable of the function

2.3 $$ \varphi ( Y ) = \sum_{i = 1}^{n} \bigl( \bigl\vert \sigma _{i} ( Y ) \bigr\vert + \varepsilon \bigr)^{p/2} = \operatorname{tr} \bigl( ( Y + \varepsilon I )^{p/2} \bigr) $$

is

$$\partial\varphi ( Y ) = \frac{p}{2}U_{1}\operatorname{Diag} \biggl\{ \frac{c_{i}}{ ( \sigma_{i} ( Y ) + \varepsilon )^{1 - \frac{p}{2}}}:i \in\Omega \biggr\} U_{1}^{T}, $$

with $Y = U_{1}\Sigma_{1}U_{1}^{T}$ being the *SVD* of *Y*, and $\Omega= \{ 1,2, \ldots,n \}$.

From (2.3), it is easier to know exactly that $\varphi ( Y )$ is concave, thus $- \varphi ( Y )$ is convex. Besides, a vector $Y^{*}$ is said to be a subgradient of a convex function *f* at a point *Y* if $f(z) \ge f(Y) + \langle Y^{*},Y - x \rangle$, for any *Z*. Therefore, based on the definition of subgradient of the convex function, we have

2.4 $$ - \varphi ( Y ) \ge- \varphi ( Y_{k} ) + \langle- G_{k},Y - Y_{k} \rangle, $$

where $- G_{k}$ is the subgradient of $- \varphi ( Y )$ at $Y_{k}$, *i.e.*, $- G_{k} \in\partial ( - \varphi ( Y_{k} ) )$. The inequality of (2.4) is equivalent to

2.5 $$ \varphi ( Y ) \le\varphi ( Y_{k} ) + \langle G_{k},Y - Y_{k} \rangle. $$

Then $\varphi ( Y_{k} ) + \langle G_{k},Y - Y_{k} \rangle$ is used as a surrogate function of $\varphi ( Y )$.

## Main results

Let $Y = X^{T}X$, then $Y = V\Sigma^{2}V^{T}$ can be obtained, where $X = U\Sigma V^{T}$ with $U \in\mathbb{R}^{m \times n}$, $V \in\mathbb{R}^{n \times n}$, and $\Sigma= \operatorname{Diag} \{ \sigma_{i} ( X ) \} \in\mathbb{R}^{n \times n}$, then $\sigma_{i} ( Y ) = ( \sigma_{i} ( X ) )^{2}$. From (2.2), (2.3), and (2.5),

3.1 $$ \operatorname{tr} \bigl( \bigl( X^{T}X + \varepsilon I \bigr)^{p/2} \bigr) \le \operatorname{tr} \bigl( \bigl( X_{k}^{T}X_{k} + \varepsilon I \bigr)^{p/2} \bigr) + \bigl\langle W_{k},X^{T}X - X_{k}^{T}X_{k} \bigr\rangle $$

can be obtained, where$W_{k} \in\frac{p}{2}V\operatorname{Diag} \{ \frac{c_{i}}{ ( ( \sigma_{i} ( X_{k} ) )^{2} + \varepsilon )^{1 - \frac{p}{2}}}:i \in\Omega \}V^{T}$.

In order to introduce the following lemma, the definitions of Lipschitz continuous of a function and the norm $\| \cdot\|_{F}$ are given, namely a function is Lipschitz continuous with constant *L* if, for any *x*, *y*, $|f(x) - f(y)| \le L\|x - y\|$; and the $\| \cdot\|_{F}$ of a matrix *X* is defined as $\|X\|_{F}: = \sqrt{\sum_{i = 1}^{m} \sum_{j = 1}^{n} x_{ij}^{2}} $.

### Lemma 3.1

([17])

*Let* $f:\mathbb{R}^{m \times n} \to \mathbb{R}$ *be a continuously differentiable function with Lipschitz continuous gradient and the Lipschitz constant* $L ( f )$. *Then*, *for any* $L \ge L ( f )$,

3.2 $$ f ( X ) \le f ( Y ) + \bigl\langle \nabla f ( Y ),X - Y \bigr\rangle + \frac{L}{2} \Vert X - Y \Vert _{F}^{2},\quad \forall X,Y \in\mathbb{R}^{m \times n}. $$

Now let $f ( X ) = \frac{1}{2} \Vert \mathrm{A} ( X ) - b \Vert _{2}^{2}$, thus the Lipschitz constant of the gradient $\nabla f ( X ) = \mathrm{A}^{\bigstar} ( \mathrm{A} ( X ) - b )$ is $L ( f ) = \lambda ( \mathrm{A}^{\bigstar} \mathrm{A} )$, where $\lambda ( \mathrm{A}^{\bigstar} \mathrm{A} )$ is the maximum eigenvalue of $\mathrm{A}^{\bigstar} \mathrm{A}$.

By using (2.1), (2.3), (3.1), and (3.2), we update $X_{k + 1}$ by minimizing the sum of these two surrogate functions

3.3 $$ \begin{aligned}[b] X_{k + 1} ={}& \arg\min\varphi \bigl( X_{k}^{T}X_{k} \bigr) + \bigl\langle W_{k},X^{T}X - X_{k}^{T}X_{k} \bigr\rangle + f ( X_{k} ) + \bigl\langle \nabla f ( X_{k} ),X - X_{k} \bigr\rangle \\ &+ \frac{L ( f )}{2} \Vert X - X_{k} \Vert _{F}^{2} \\ ={}& \arg\min\tau \bigl\langle W_{k},X^{T}X \bigr\rangle + \frac{\rho}{ 2} \biggl\Vert X - \biggl( X_{k} - \frac{1}{\rho} \nabla f ( X_{k} ) \biggr) \biggr\Vert _{F}^{2}, \end{aligned} $$

where $\rho\ge\frac{L ( f )}{2}$.

### Lemma 3.2

*If the function* $g ( X ) = \langle Q,X^{T}X \rangle$ *with* $X \in\mathbb{R}^{m \times n}$ *and* $Q \in\mathbb{R}^{n \times n}$, *then the gradient of* $g ( X )$ *is* $\nabla g ( X ) = 2XQ$.

### Proof

Consider the auxiliary function $\theta:\mathbb{R} \to \mathbb{R}$, given by $\theta ( t ) = g ( X + tY )$, for any arbitrary matrix $Y \in\mathbb{R}^{m \times n}$. From the basic calculus, it can be known that $\theta' ( 0 ) = \langle \nabla g ( X ),Y \rangle$. By the definition of the derivative of function, it follows that

3.4 $$ \begin{aligned}[b] \theta' ( 0 )& = \lim _{t \to0}\frac{\theta ( t ) - \theta ( 0 )}{t} = \lim_{t \to 0} \frac{g ( X + tY ) - g ( X )}{t} \\ &= \lim_{t \to0}\frac{ \langle Q, ( X + tY )^{T} ( X + tY ) \rangle- \langle Q,X^{T}X \rangle}{t} \\ &= \lim_{t \to0}\frac{t \langle Q,X^{T}Y \rangle+ t \langle Q,Y^{T}X \rangle+ t^{2} \langle Q,Y^{T}Y \rangle}{t} \\ &= \bigl\langle Q,X^{T}Y \bigr\rangle + \bigl\langle Q,Y^{T}X \bigr\rangle = \operatorname{tr} \bigl( Q^{T}X^{T}Y \bigr) + \operatorname{tr} \bigl( Y^{T}XQ \bigr) = \langle2XQ,Y \rangle \end{aligned} $$

thus the gradient of $g ( X )$ is $\nabla g ( X ) = 2XQ$. □

Based on the above analysis, this paper proposes the following algorithm.

### Algorithm 1

(Proximal iteratively reweighted algorithm to solve problem (2.1))

*1*: *Input*: $\rho\ge\frac{L ( f )}{2}$, where $L ( f )$ is the Lipschitz constant of $f ( x )$.
*2*: *Initialization*: $k = 0$, $W_{k}$.
*3*: Update $X^{k + 1}$ by solving the following problem:
  $$X_{k + 1} = \arg\min\tau \bigl\langle X^{T}X,W_{k} \bigr\rangle + \frac{\rho}{2} \bigg\Vert X - \biggl( X_{k} - \frac{1}{\rho} \mathrm {A}^{\bigstar} \bigl( \mathrm{A} ( X_{k} ) - b \bigr) \biggr) \bigg\Vert _{F}^{2}.$$
*4*: Update the weight $W_{k + 1}$ by
  $$W_{k + 1} \in- \partial \bigl( - \varphi ( Y_{k + 1} ) \bigr),\quad\text{where } Y_{k + 1} = X_{k}^{T}X_{k}.$$
*5*: Output low-rank matrix $X^{k}$.

### Theorem 3.3

*Let* $\rho\ge\frac{L ( f )}{2}$, *where* $L ( f )$ *is the Lipschitz constant of* $f ( x )$. *The sequence* $\{ X_{k} \}$ *generated in Algorithm* 1 *satisfies*

1. $$F ( X_{k} ) - F ( X_{k + 1} ) \ge \biggl( \rho- \frac{L ( f )}{2} \biggr) \Vert X_{k} - X_{k + 1} \Vert _{F}^{2}. $$
2. *The sequence* $\{ X_{k} \}$ *is bounded*.
3. $\sum_{k = 1}^{\infty} \Vert X_{k} - X_{k + 1} \Vert _{F}^{2} < \frac{2\theta}{2\rho- L ( f )}$. *In particular*, $\lim_{k \to\infty} \Vert X_{k} - X_{k + 1} \Vert _{F} = 0$.

### Proof

Since $X_{k + 1}$ is the globally optimal solution of problem (3.3), and the zero matrix is contained in the subgradient with respect to *X*. That is, there exists a matrix $X_{k + 1} \in$ such that

3.5 $$ 2\tau X_{k + 1}W_{k} + \nabla f ( X_{k} ) + \rho ( X_{k + 1} - X_{k} ) = 0. $$

By using the above equality of (3.4) and (3.5), we get

3.6 $$ 2\tau \langle X_{k + 1}W_{k},X_{k + 1} - X_{k} \rangle+ \bigl\langle \nabla f ( X_{k} ),X_{k + 1} - X_{k} \bigr\rangle + \rho \Vert X_{k + 1} - X_{k} \Vert _{F}^{2} = 0. $$

Since the function $\langle W_{k},X^{T}X \rangle$ is a convex function on *X*, thus

$$\bigl\langle W_{k},X_{k}^{T}X_{k} \bigr\rangle - \bigl\langle W_{k},X_{k + 1}^{T}X_{k + 1} \bigr\rangle \ge2 \langle X_{k + 1}W_{k},X_{k} - X_{k + 1} \rangle, $$

and the above equality also can be rewritten as

3.7 $$ \bigl\langle W_{k},X_{k}^{T}X_{k} - X_{k + 1}^{T}X_{k + 1} \bigr\rangle \ge2 \langle X_{k + 1}W_{k},X_{k} - X_{k + 1} \rangle. $$

Then it follows from (3.6) and (3.7) that

3.8 $$ \tau \bigl\langle W_{k},X_{k}^{T}X_{k} - X_{k + 1}^{T}X_{k + 1} \bigr\rangle \ge- \bigl\langle \nabla f ( X_{k} ),X_{k} - X_{k + 1} \bigr\rangle + \rho \Vert X_{k + 1} - X_{k} \Vert _{F}^{2}. $$

Let $f ( X ) = \frac{1}{2} \Vert \mathrm{A} ( X ) - b \Vert _{2}^{2}$, and according to Lemma 3.1,

3.9 $$ f ( X_{k} ) - f ( X_{k + 1} ) \ge \bigl\langle \nabla f ( X_{k} ),X_{k} - X_{k + 1} \bigr\rangle - \frac{L ( f )}{2} \Vert X_{k} - X_{k + 1} \Vert _{F}^{2} $$

can be obtained. Since the function $\operatorname{tr} ( ( X^{T}X + \varepsilon I )^{p/2} )$ is concave, and just like (3.1), then it can be obtained

3.10 $$ \operatorname{tr} \bigl( \bigl( X_{k}^{T}X_{k} + \varepsilon I \bigr)^{p/2} \bigr) - \operatorname{tr} \bigl( \bigl( X_{k + 1}^{T}X_{k + 1} + \varepsilon I \bigr)^{p/2} \bigr) \ge \bigl\langle W_{k},X_{k}^{T}X_{k} - X_{k + 1}^{T}X_{k + 1} \bigr\rangle . $$

Now, combining (3.8), (3.9), and (3.10), we get

$$\begin{aligned} F ( X_{k} ) - F ( X_{k + 1} ) &= \tau \operatorname{tr} \bigl( \bigl( X_{k}^{T}X_{k} + \varepsilon I \bigr)^{p/2} \bigr) + f ( X_{k} ) - \tau \operatorname{tr} \bigl( \bigl( X_{k + 1}^{T}X_{k + 1} + \varepsilon I \bigr)^{p/2} \bigr) - f ( X_{k + 1} ) \\ &\ge \biggl( \rho- \frac{L ( f )}{2} \biggr) \Vert X_{k + 1} - X_{k} \Vert _{F}^{2} \ge0. \end{aligned} $$

Thus, $F ( X_{k} )$ is monotonically decreasing. Given the facts of all inequalities above for $k \ge1$, it can be obtained

3.11 $$ F ( X_{1} ) - F ( X_{k + 1} ) \ge \biggl( \rho- \frac{L ( f )}{2} \biggr)\sum_{i = 1}^{k} \Vert X_{i + 1} - X_{i} \Vert _{F}^{2}, $$

and from (3.11) it follows that

3.12 $$ \biggl( \rho- \frac{L ( f )}{2} \biggr)\sum_{i = 1}^{k} \Vert X_{i + 1} - X_{i} \Vert _{F}^{2} \le F ( X_{1} ) < + \infty. $$

Then, for $k \to\infty$, (3.12) implies that

$$\lim_{k \to\infty} \Vert X_{k + 1} - X_{k} \Vert _{F} = 0. $$

Since the objective function $F ( X )$ in problem (2.1) is nonnegative and satisfies

$$F ( X ) \to\infty,\quad \text{as }\Vert X \Vert _{F} \to\infty, $$

then $X_{k} \in \{ X:0 \le F ( X ) \le F ( X_{1} ) \}$ and the sequence $\{ X_{k} \}$ is bounded.

Therefore, the proof has been completed. □

### Theorem 3.4

*Let* $\{ X_{k} \}$ *be the sequence generated in Algorithm* 1. *Then any accumulation point of* $\{ X_{k} \}$ *is a stationary point* $X^{\bigstar} $ *of the problem*. *Moreover*, *for* $k = 1,2, \ldots,N$, *there always exists*

$$\min_{1 \le k \le N} \Vert X_{k + 1} - X_{k} \Vert _{F}^{2} \le \frac{F ( X_{1} ) - F ( X^{\bigstar} )}{n ( \rho- \frac{L ( f )}{2} )}. $$

### Proof

Since the sequence $\{ X_{k} \}$ generated in Algorithm 1 is bounded, there exist an accumulation point $X^{\bigstar} $ and a subsequence $\{ X_{kj} \}$ such that $\lim_{j \to\infty} X_{kj} = X^{\bigstar} $. Assume that $X_{kj}$ is the solution of problem (3.3), it can be obtained

$$2\tau X_{kj + 1}W_{kj} + \nabla f ( X_{kj} ) + \rho ( X_{kj + 1} - X_{kj} ) = 0. $$

Let $j \to\infty$, according to Theorem 3.3, $\lim_{j \to\infty} \Vert X_{kj + 1} - X_{kj} \Vert _{F} = 0$ can be obtained. Hence, there exists the matrix

$$W^{\bigstar} = \frac{p}{2}V_{2}\operatorname{Diag} \biggl\{ \frac{1}{ ( ( \sigma_{i} ( X^{\bigstar} ) )^{2} + \varepsilon )^{1 - \frac{p}{2}}} \biggr\} V_{2}^{T} = \frac{p}{2} \bigl( \bigl( X^{\bigstar} \bigr)^{T}X^{\bigstar} + \varepsilon I \bigr)^{p/2 - 1}, $$

where $X^{\bigstar} = U_{2}\Sigma_{2}V_{2}^{T}$ with $U_{2} \in\mathbb{R}^{m \times n}$, $V_{2} \in\mathbb{R}^{n \times n}$, and $\sum= \operatorname{Diag} \{ \frac{1}{ ( ( \sigma_{i} ( X^{\bigstar} ) )^{2} + \varepsilon )^{1 - \frac{p}{2}}} \}$.

By the above analysis, it can be known that

$$\tau\rho X^{\bigstar} \bigl( \bigl( X^{\bigstar} \bigr)^{T}X^{\bigstar } + \varepsilon I \bigr)^{p/2 - 1} + \nabla f ( X_{\bigstar} ) = 0, $$

then $X^{\bigstar} $ is a stationary point of problem (2.1).

Moreover, by using (3.11), for $k = 1,2, \ldots,N$, it can be obtained

$$\begin{aligned} F ( X_{1} ) - F ( X_{N + 1} ) &\ge \biggl( \rho- \frac{L ( f )}{2} \biggr)\sum_{k = 1}^{N} \Vert X_{k + 1} - X_{k} \Vert _{F}^{2} \\ &\ge N \biggl( \rho- \frac{L ( f )}{2} \biggr)\min_{1 \le k \le N} \Vert X_{k + 1} - X_{k} \Vert _{F}^{2}. \end{aligned} $$

Thus

$$\begin{aligned} \min_{1 \le k \le N} \Vert X_{k + 1} - X_{k} \Vert _{F}^{2} &\le\frac{F ( X_{1} ) - F ( X_{N + 1} )}{n ( \rho- \frac{L ( f )}{2} )} \\ &\le\frac{F ( X_{1} ) - F ( X^{\bigstar} )}{n ( \rho- \frac{L ( f )}{2} )} \end{aligned} $$

can be obtained, which completes the proof. □

## Conclusion

A proximal iteratively reweighted algorithm based on the weighted fixed point method for recovering a low-rank matrix problem has been presented in this paper. Due to the special properties of the nonconvex surrogate function, the algorithm in this study iteratively has a closed form solution to solving a weighted singular value thresholding problem. Finally, it has been proved that the algorithm can decrease the objective function value monotonically and any limit point is a stationary point.
